# Insight into the Mechanisms of Adenovirus Capsid Disassembly from Studies of Defensin Neutralization

**DOI:** 10.1371/journal.ppat.1000959

**Published:** 2010-06-24

**Authors:** Jason G. Smith, Mariena Silvestry, Steffen Lindert, Wuyuan Lu, Glen R. Nemerow, Phoebe L. Stewart

**Affiliations:** 1 Department of Immunology and Microbial Science, The Scripps Research Institute, La Jolla, California, United States of America; 2 Department of Molecular Physiology and Biophysics, Vanderbilt University Medical Center, Nashville, Tennessee, United States of America; 3 Institute of Human Virology, University of Maryland School of Medicine, Baltimore, Maryland, United States of America; North Carolina State University, United States of America

## Abstract

Defensins are effectors of the innate immune response with potent antibacterial activity. Their role in antiviral immunity, particularly for non-enveloped viruses, is poorly understood. We recently found that human alpha-defensins inhibit human adenovirus (HAdV) by preventing virus uncoating and release of the endosomalytic protein VI during cell entry. Consequently, AdV remains trapped in the endosomal/lysosomal pathway rather than trafficking to the nucleus. To gain insight into the mechanism of defensin-mediated neutralization, we analyzed the specificity of the AdV-defensin interaction. Sensitivity to alpha-defensin neutralization is a common feature of HAdV species A, B1, B2, C, and E, whereas species D and F are resistant. Thousands of defensin molecules bind with low micromolar affinity to a sensitive serotype, but only a low level of binding is observed to resistant serotypes. Neutralization is dependent upon a correctly folded defensin molecule, suggesting that specific molecular interactions occur with the virion. CryoEM structural studies and protein sequence analysis led to a hypothesis that neutralization determinants are located in a region spanning the fiber and penton base proteins. This model was supported by infectivity studies using virus chimeras comprised of capsid proteins from sensitive and resistant serotypes. These findings suggest a mechanism in which defensin binding to critical sites on the AdV capsid prevents vertex removal and thereby blocks subsequent steps in uncoating that are required for release of protein VI and endosomalysis during infection. In addition to informing the mechanism of defensin-mediated neutralization of a non-enveloped virus, these studies provide insight into the mechanism of AdV uncoating and suggest new strategies to disrupt this process and inhibit infection.

## Author Summary

Defensins are effectors of the innate immune response with antibacterial and antiviral activity. A major bactericidal mechanism of defensins is membrane disruption; however, their mechanism against non-enveloped viruses, such as human adenovirus, is poorly understood. This work shows that sensitivity of human adenovirus to defensins is species specific and that neutralization is dependent upon defensin tertiary structure. A cryoEM structural study of an adenovirus vector in complex with a neutralizing defensin, HD5, led to a neutralization model in which defensin binds to the interface of two capsid proteins, preventing dissociation of the fiber protein. We propose that binding at this site blocks downstream uncoating events required for infection. Infectivity studies using virus chimeras comprised of capsid proteins from sensitive and resistant human adenovirus serotypes support this model. This functional and structural study provides insight into the mechanism of human adenovirus neutralization by defensins and suggests new strategies to inhibit infection.

## Introduction

Defensins are an evolutionarily conserved family of antimicrobial peptides that are an important effector component of the innate immune response. Humans express two classes of defensins, α- and β-defensins. There are six human α-defensins (HNP1–4, HD5, and HD6) and multiple β-defensins, which differ in their tissue distribution and expression patterns [1], [2]. Both α- and β-defensins are small peptides with three intramolecular disulfide bonds and are potent antibacterial agents. There is substantial evidence that a major bactericidal mechanism of defensins is through membrane disruption [3], and lipid bilayer interactions are facilitated by their amphipathicity and net positive charge. A growing body of evidence suggests that certain defensins are also potent antivirals. For enveloped viruses, direct disruption of the viral lipid envelope bilayer has been proposed as a mechanism for neutralization [4]. In addition, several defensins have been shown to be lectins and to block human immunodeficiency virus and Herpes simplex virus binding to cellular receptors [5]–[7].

Defensins have also been shown to neutralize several non-enveloped viruses, including human adenovirus (HAdV), human papillomavirus (HPV), adeno-associated virus (AAV), and polyomavirus, despite the absence of a lipid target [8]–[14]. We have chosen HAdV as a tractable model system to analyze this process at the molecular level. AdV is a dsDNA virus with an icosahedral capsid composed primarily of 240 trimers of hexon. Each of the twelve icosahedral vertices contains a penton complex comprised of the non-covalently associated fiber and penton base proteins. The capsid is stabilized by proteins IIIa, VI, VIII, and IX. There are 52 serotypes of HAdV divided into 7 species, A–G [15], [16]. Three additional types (HAdV-53, -54, and -55) have also recently been described [17], [18]. The mode of cell entry is best understood for the HAdV-C serotypes in cultured epithelial cells and is initiated by a high affinity interaction between the distal knob of the fiber and one of several cell surface receptors [19]. Internalization via clathrin-mediated endocytosis is triggered by the interaction between an RGD motif in penton base and cellular integrin co-receptors [20]. Uncoating, which is the removal of the protective protein shell from the viral genome, occurs in a stepwise fashion beginning with dissociation of the fiber from the capsid at or near the cell surface [21], [22]. Additional uncoating events, including release of the endosomalytic protein VI, occur in the endosome in response to cellular triggers such as acidification [23]. Upon escape from the early endosome, the partially uncoated capsid travels along microtubules and docks at the nuclear pore complex, where the viral genome enters the nucleus [24].

Our previous studies revealed the stage in the virus entry pathway that is blocked by defensins [13]. We found that the α-defensins HNP1 and HD5 significantly inhibit HAdV-5 infection at low micromolar concentrations. Although receptor binding and virus internalization were unaffected, virus escape from the endosome was blocked. Moreover, defensin binding stabilized the virus capsid in thermal denaturation assays. These observations are consistent with a mechanism by which defensins neutralize AdV infection by blocking uncoating and release of the endosomalytic protein VI. We have now extended these studies to determine the specificity of defensin binding to HAdV, to approximate the stoichiometry and affinity of this interaction, and to identify the neutralization determinants on the virus capsid. These studies not only contribute to an understanding of the mechanism of defensin-mediated neutralization of non-enveloped virus infection but also provide insight into the process of HAdV uncoating during infection.

## Results

### Sensitivity of HAdV to defensins is species specific

**Figure 1:**
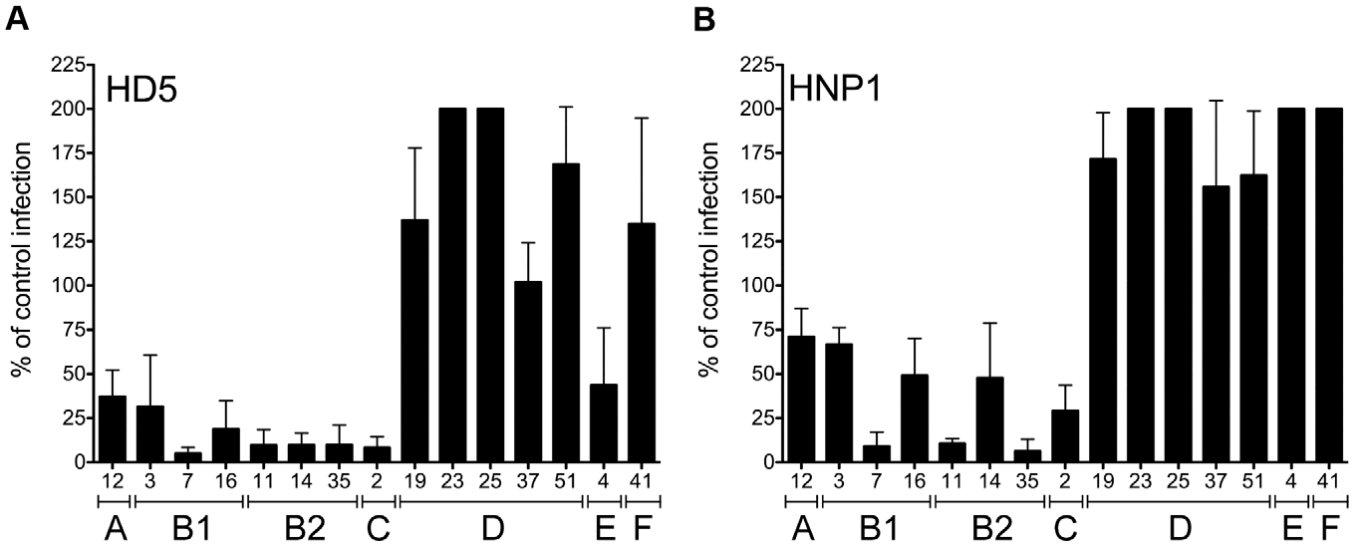
Sensitivity to neutralization by alpha-defensins is AdV species specific. HAdVs were incubated with 15 µM HD5 (A) or HNP1 (B) and assessed for infectivity on A549 cells. Data are the mean of the percent infectivity compared to control cells infected with each virus in the absence of defensin for at least three independent experiments ± SD. The upper limit for quantification of this assay is 200%. Virus serotypes are grouped by species (A–F).

In our previous studies we showed that a small subset of HAdVs, including HAdV-5, -12, and -35 (species C, A, and B2, respectively) are neutralized by α-defensins [13]; however, the underlying molecular mechanisms were not delineated. To determine whether sensitivity to α-defensins is a general property of HAdVs, serotypes representative of HAdV species A–F were tested for infectivity in the presence of 15 µM HD5 or HNP1 (Figure 1). Wild type HAdVs rather than vectors were used for these studies, and infectivity was assessed by staining for hexon production. We found that each of HAdV types belonging to species A, B1, B2, C, and E is sensitive to HD5. Strikingly, the HAdV-D and F serotypes are completely resistant to HD5-mediated neutralization and, in most cases, infection is actually enhanced. Serotypes sensitive to HD5 are also generally sensitive to HNP1, although only modest inhibition was observed for HAdVs-3, -12, -14, and -16. One exception is HAdV-4 (species E), which is moderately sensitive to HD5 but resistant to HNP1. None of the tested serotypes is sensitive to the β-defensin HBD2 (data not shown). These studies indicate that sensitivity to α-defensins is species specific. In addition, particular HAdV serotypes are not equally sensitive to all defensins, indicating defensin sequence specificity as well.

### HAdV neutralization correlates with defensin binding to the capsid

**Figure 2:**
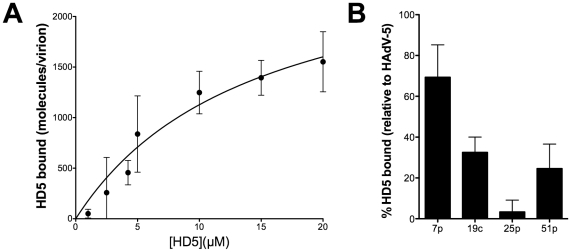
HD5 binding to HAdV. A) HD5 binding to a representative defensin-sensitive serotype (HAdV-5) was quantified by an equilibrium-binding assay. Data are the mean of the number of HD5 molecules bound per virion at the indicated HD5 concentrations from at least three independent experiments ± SD. Binding curves were fitted using Prism software. B) Binding of HD5 to additional defensin-sensitive (HAdV-7p) and resistant (HAdV-19c, -25p, -51p) serotypes expressed as a percent of HD5 bound to HAdV-5. Data are the mean of two or three independent experiments ± SD.

Neutralization of HAdV by α-defensins is dependent upon binding to the virus capsid, which can be disrupted in the presence of elevated concentrations of sodium chloride [13]. To perform a more quantitative assessment of this interaction, increasing concentrations of HD5 were incubated with Ad5.eGFP to allow binding, and the virus/HD5 complex was then separated from unbound HD5 on a nycodenz gradient. Bound defensin was visualized after SDS-PAGE using a sensitive fluorescent total protein stain and quantified against a standard curve. These experiments showed that defensin binding to the HAdV-5 capsid is saturable, suggesting specificity, and that at saturation approximately 2750 HD5 molecules are bound to each virus particle (95% confidence interval = 1603–3919 HD5 molecules) (Figure 2A). In addition, the K_D_ of this interaction is approximately 14.5 µM (95% confidence interval = 2.8–26.2 µM), which correlates reasonably well with the IC_50_ of HD5 for HAdV-5 infection (3–4 µM) [13].

We assessed HD5 binding to additional serotypes after incubation with 20 µM HD5 (Figure 2B). The sensitive serotype HAdV-7p bound 69.3±15.9% of the amount of HD5 bound to HAdV-5 in parallel samples; whereas, the corresponding values for the resistant serotypes HAdV-19c, -25p, and -51p were 32.5±7.5%, 3.3±5.8%, and 24.6±12.0%, respectively. These studies demonstrate reduced binding of HD5 to resistant serotypes, suggesting that binding of α-defensins to species-specific features on the HAdV capsid correlates with neutralization.

### Neutralization by HD5 is dependent upon defensin tertiary structure

**Figure 3:**
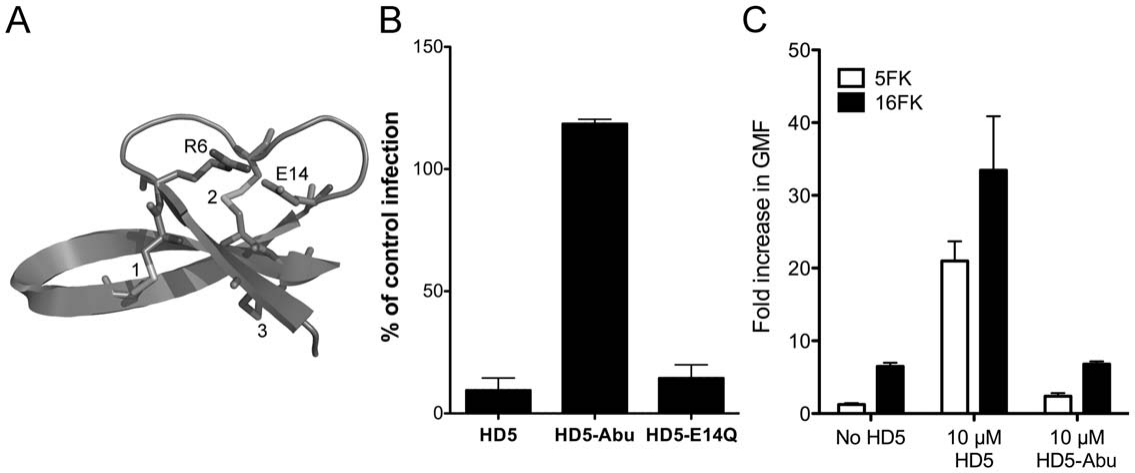
HD5 antiviral activity is structure dependent. A) Ribbon representation of HD5 (PDB 1ZMP). Three disulfide bonds are numbered, and the two residues comprising the conserved salt bridge (R6 and E14) are indicated. B) Ad5.eGFP was incubated with 15 µM of each of the indicated defensins. HD5-Abu is HD5 with the six cysteines replaced with L-α-aminobutyric acid. Data are the mean percent of eGFP positive cells compared to control cells infected in the absence of defensin for at least three independent experiments ± SD. C) Binding of Alexa Fluor 488-labeled HAdV-5 to cells was assessed after incubation with or without 20 µM HD5 or HD5-Abu and in competition with 100 nM 5FK or 16 FK. Data are the mean fold increase in geometric mean fluorescence compared to cells alone and are of at least 10,000 cells from each of three independent experiments ± SD.

To gain further insight into the defensin-HAdV interaction, we assessed the requirement for two conserved structural elements on the anti-AdV activity of HD5. All defensins have three disulfide bonds (Figure 3A). In addition, all α-defensins possess a conserved salt bridge, such as that comprised of glutamic acid 14 (E14) and arginine 6 (R6) in HD5, which has been shown to increase defensin protease resistance [25], [26]. The antibacterial properties of defensins, which are dependent upon protein-lipid interactions, are not uniformly conformation dependent [27]–[29]. In some cases, incorrectly folded analogs are more potent antibacterial agents than the correctly folded defensin molecule. In contrast, defensin-related chemokine activity, which relies on protein-protein interactions, is dependent on defensin conformation [28]. We hypothesized that the defensin-capsid interaction for sensitive serotypes would likely be dependent upon defensin conformation, as this would be more typical for protein-protein interactions. To test this hypothesis, HD5 derivatives in which the six cysteines were replaced with L-α-aminobutyric acid (HD5-Abu), to prevent the formation of disulfide bonds, or containing a substitution of glutamine for glutamic acid 14 (HD5-E14Q), to disrupt the conserved salt bridge, were tested for their activity against Ad5.eGFP (Figure 3B). Disruption of the R6-E14 salt bridge had no effect on antiviral activity. In contrast, HD5-Abu failed to inhibit Ad5.eGFP infection, and no detectable binding of HD5-Abu was observed upon incubation at 20 µM with HAdV-5 (data not shown). Therefore, HAdV neutralization does not merely require an amphipathic molecule with a net positive charge. Rather, specific interactions mediated by the correctly folded α-defensin molecule are required.

### Binding of HD5 to HAdV-5 does not block receptor interaction

Previously we observed that HD5 enhances binding of HAdV-5 to cells despite an almost complete block to productive infection [13]. In order to determine the receptor-dependence of this effect, we pre-incubated cells with recombinant fiber knob from HAdV-5 (5FK) to block receptor (CAR) interactions and measured cell binding of fluorescently labeled HAdV-5 that was pre-incubated with or without HD5 or HD5-Abu (Figure 3C). Cells incubated with fiber knob from HAdV-16 (16FK), which binds CD46, served as a control. We observed that virus binding to cells was reduced 5.2-fold in the presence of 5FK compared to 16FK. This confirms the receptor-dependence of the normal interaction of HAdV-5 with cells. Pre-incubation with HD5 increased virus binding to cells. In this case the presence of 5FK reduced binding 1.6-fold compared to 16FK, indicating some receptor-dependence of the virus/cell interaction even in the presence of HD5. Virus pre-incubated with HD5-Abu was equivalent to virus alone, consistent with the failure of HD5-Abu to bind to virus. These studies confirm that HD5 binding to HAdV-5 does not completely block the interaction with CAR receptor.

### CryoEM structural analysis of a HAdV-HD5 complex

**Figure 4:**
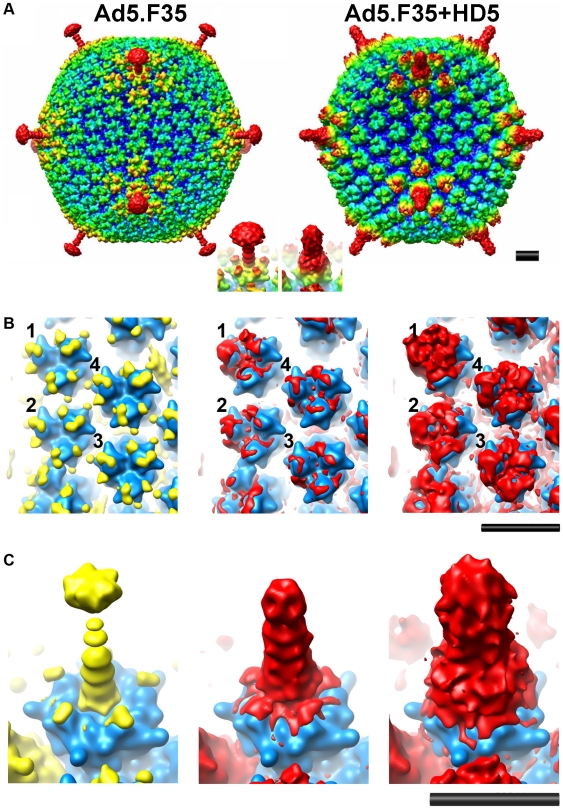
CryoEM structures of Ad5.F35 and Ad5.F35+HD5. A) Reconstructions viewed along icosahedral 2-fold axes and shown radially color-coded (blue = 405 Å; cyan = 425 Å; green = 445 Å, yellow = 465 Å; red = 485 Å). Inset, enlarged views of the vertex regions. B) and C) Ad5.F35 and Ad5.F35+HD5 difference maps. The density representations of the docked hexon and penton base/fiber complex are in blue, the Ad5.F35 difference map is in yellow, and the Ad5.F35+HD5 difference map is in red. Two threshold levels are shown for the Ad5.F35+HD5 difference map, one showing only the strongest density (middle) and a second at just above the noise level (right). Only one threshold level is shown for the Ad5.F35 difference map at just above the noise level. B) Four unique hexons, numbered 1–4, within the asymmetric unit of the icosahedral capsid. C) Penton base and fiber viewed at a 45° angle. Scale bars, 100Å.

To obtain structural insight into the mechanism of α-defensin-mediated neutralization of HAdV infection, we studied a complex of Ad5.F35 (Ad35F) and HD5 by cryoelectron microscopy (cryoEM). This chimeric virus construct was chosen because of its short fiber and the availability of a cryoEM structure of Ad5.F35 in the absence of defensin for comparison [30], [31]. The sensitivity of Ad5.F35 to HD5 is comparable to that of HAdV-5 and HAdV-35 (data not shown). Ad5.F35 was incubated with a saturating concentration of HD5 (20 µM) then applied to grids and flash frozen for cryoEM. A dataset of 2,611 cryoEM particle images of the Ad5.F35+HD5 complex was collected and processed as performed earlier for Ad5.F35 [31]. The resolution of the icosahedral portion of the Ad5.F35+HD5 reconstruction is estimated as 12 Å by the FSC 0.5 threshold criterion, compared to 6.9 Å for the Ad5.F35 reconstruction. Both cryoEM structures are shown filtered to 12 Å resolution in Figure 4A. The most noticeable difference between the two structures is the presence of more density on top of penton base and around the fiber shaft in the Ad5.F35+HD5 structure. In addition, while the fiber knob is visible in the Ad5.F35 structure, it is only weakly reconstructed in the Ad5.F35+HD5 structure (Figure 4A inset).

In order to identify the binding regions for HD5 on the surface of Ad5.F35, we performed a difference map analysis using the available crystal structure of the HAdV-5 hexon (PDB 1P30) [32] and the co-crystal structure of HAdV-2 penton base bound to a peptide derived from the N-terminus of fiber (PDB 1X9T) [33]. Difference mapping with the crystal structures was preferable to direct subtraction of the Ad5.F35 structure from the Ad5.F35+HD5 structure because of ringing in the cryoEM density maps due to incomplete correction for the contrast transfer function of the microscope. There are multiple flexible loops with a total of 51 residues per monomer at the top of hexon (hexon towers) that are missing from the crystal structure. Density for these loops (yellow) is clearly visible in the Ad5.F35 difference map and nearly identical for each of the four unique hexons within the icosahedral asymmetric unit (Figure 4B, left panel). The Ad5.F35+HD5 difference map shows density (red) on the hexon towers that is attributable to both HD5 and the missing hexon loops (Figure 4B, middle and right panels). This density is variable for each unique hexon and greatest above the peripentonal hexon (position 1). The variability in the Ad5.F35+HD5 difference map suggests that HD5 interacts with and induces additional conformational heterogeneity in the flexible loops of hexon. HD5 difference density is also found within the central depression of the hexon trimers in the same location identified for binding of Factor X [34], [35]. This central depression contains multiple negatively charged residues that are likely to form a binding site for the positively charged HD5 molecule.

The difference map analysis reveals multiple binding sites for HD5 on the penton complex (penton base and fiber). The Ad5.F35 difference map clearly reveals the flexible RGD loop of penton base (78 residues) as well as the shaft and knob of fiber (yellow), which are missing from the penton base/fiber peptide co-crystal structure (Figure 4C, left panel). The Ad5.F35+HD5 difference map shows significant additional density attributable to HD5 (red) on top of the penton base and around the fiber shaft (Figure 4C, middle and right panels). The conformation of the flexible RGD loop of penton base appears to be perturbed in the presence of HD5. The position of the fiber knob relative to the fiber shaft also seems to be modified such that the knob is no longer reconstructed.

### Model for HD5 neutralization of HAdV

**Figure 5:**
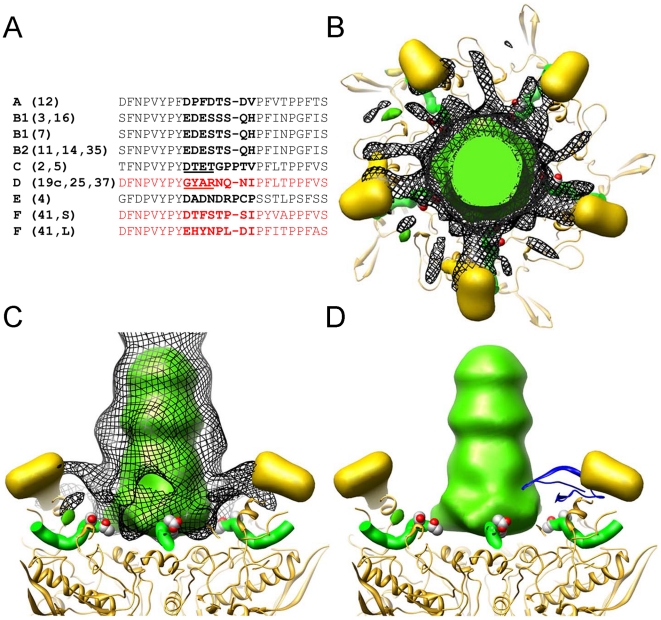
HD5 binding regions on the penton base and fiber. (A) Alignment of the N-terminal sequences of fiber from serotypes that were studied for defensin sensitivity. Several sequences are representative of multiple serotypes within a species, as indicated. HAdV-F serotypes have a short (S) and long (L) fiber. No sequence information is available for this region for HAdV-23 and -51. Sequences for defensin-resistant serotypes are in red and those for defensin-sensitive serotypes are in black. The variable region is in bold with the key residues underlined for HAdV-C and HAdV-D. Sequences shown correspond to residues 10 to 35 in HAdV-2. B-D) The strongest density in the Ad5.F35+HD5 difference map (black mesh) is shown together with the Ad5.F35 difference map (colored in gold for the RGD loop of the penton base and green for the fiber shaft). Also shown are the docked penton base (gold ribbon) and N-terminal fiber peptide (green ribbon) from the HAdV-2 penton crystal structure (PDB 1X9T). The side chains of fiber residues Asp-18 and Thr-19 (DT of DTET) are shown in a space filling representation. The penton complex is shown in both top (B) and side (C) views. In (D), the crystal structure of an HD5 monomer (PDB 1ZMP, blue ribbon) is included for scale.

The cryoEM analysis of the Ad5.F35+HD5 complex indicates that HD5 interacts with the exposed surfaces of the three major capsid proteins: hexon, penton base, and fiber. Previously, we observed that HD5 does not prevent HAdV-5 from entering host cells [13], [36]. In addition, we observed that HD5 binding stabilizes the capsid and prevents dissociation of capsid proteins, including fiber, upon exposure to heat. Therefore, we considered which of the multiple binding sites visualized by cryoEM might lead to enhanced virion stability. We reasoned that we might find a negatively charged region of the capsid that was present within the protein sequences of sensitive serotypes and not present in resistant serotypes and that was also in the vicinity of HD5 cryoEM difference density. In particular, we were looking for a possible binding site for HD5 that might bridge adjacent capsid subunits and stabilize the capsid. By comparing the N-terminal sequences of fibers from HAdV types that are either sensitive or resistant to HD5 we identified one negatively charged region that is present in all of the sensitive serotypes (i.e., 18-DTET-21 in HAdV-5; DPFD in HAdV-12; EDES in HAdV-3; DADN in HAdV-4) (Figure 5A). In resistant serotypes of species D the corresponding region of the fiber is non-polar and positively charged (i.e., 18-GYAR-21 in HAdV-19c). Serotype HAdV-41 is resistant to HD5, despite having a single negatively charged residue in this region; however, it is different from all of the other serotypes we examined in that it has both a short and a long fiber, which could affect the mechanism of HD5 neutralization. The variable fiber region, which is conserved within species but varies between species, precedes the fiber shaft repeats and directly follows a conserved motif (FNPVYPY) that binds at the interface of adjacent penton base monomers [33]. The difference density analysis of Ad5.F35+HD5 (Figure 5B and C) suggests a possible explanation for fiber stabilization by HD5, as strong difference density (mesh) appears to cover the variable fiber sequence (EDES in HAdV-35 or DTET in HAdV-2, partially shown in space filling representation), effectively pinning the N-terminus of fiber (green ribbon) against penton base (gold ribbon). Therefore, this variable region of the fiber may form part of a critical binding site for HD5 neutralization of HAdV. We propose a model for HD5 neutralization of HAdV-5 in which HD5 binds to the interface of penton base and fiber and prevents fiber dissociation, consequently blocking downstream uncoating events that are required for infection.

### Fiber and penton base proteins contain critical neutralization determinants

**Figure 6:**
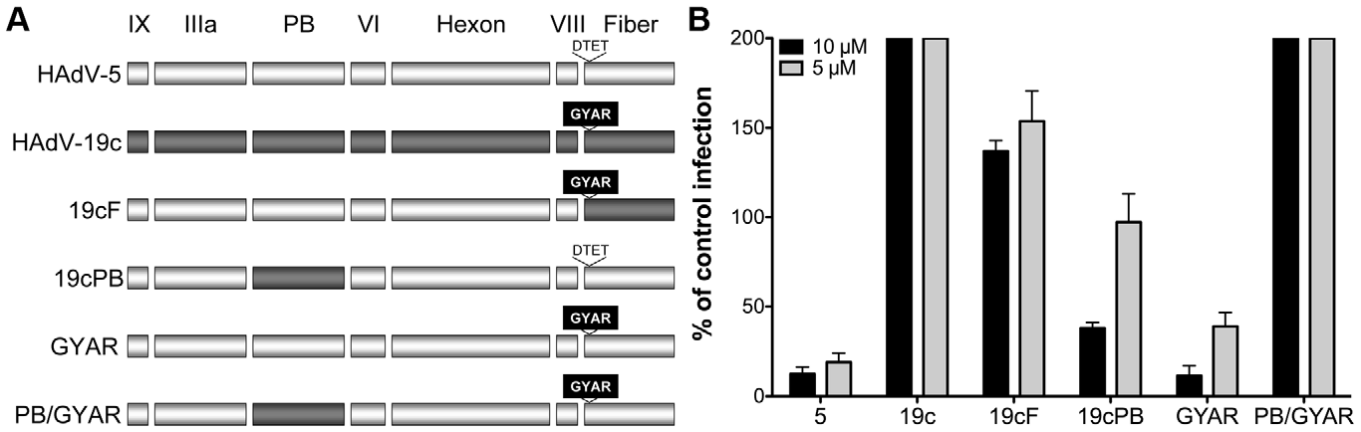
Neutralization determinants are located in fiber and penton base. A) Schematic of chimeric viruses. Capsid proteins are depicted in the order in which they are encoded in the virus genome for HAdV-5 (white) and HAdV-19c (grey). The variable residues in fiber (GYAR and DTET) are indicated for each construct. B) Each of the chimeric viruses was incubated with 5 µM (grey) or 10 µM (black) HD5 and assessed for infectivity on A549 cells. Data are the mean of the percent infectivity compared to control cells infected with each virus in the absence of defensin for at least three independent experiments ± SD. The upper limit for quantification of this assay is 200%.

The availability of sensitive (e.g., HAdV-5) and resistant (e.g., HAdV-19c) serotypes provided a means to test this HD5 neutralization model by generating virus chimeras. Initially, virus chimeras were constructed by replacing the sequences for fiber, penton base, and hexon in the HAdV-5 genome with the corresponding sequences from HAdV-19c. Consistent with previous studies [37], [38], the virus chimera containing the HAdV-19c hexon is not viable; however, constructs containing the HAdV-19c fiber (19cF) or penton base (19cPB) are capable of replicating. When each of these viruses was tested for sensitivity to HD5, we found that the 19cF virus is completely resistant to neutralization (Figure 6). In contrast, the 19cPB virus has an intermediate phenotype. It is partially neutralized by HD5 but only at the higher concentration tested (10 µM). Together, these results indicate that both fiber and penton base are involved in HD5 neutralization.

Based on these results, we created an additional construct in which only the four residues in the HAdV-5 fiber variable region (DTET) were replaced by the corresponding residues from HAdV-19c (GYAR). Compared to HAdV-5, the GYAR virus is less sensitive to 5 µM HD5. We then combined the GYAR substitution with the PB substitution in a single construct (PB/GYAR). This construct, like 19cF and wild type HAdV-19c, is completely resistant to neutralization by HD5. This result confirms a role for the DTET/GYAR variable fiber region in HD5 neutralization, as the PB/GYAR chimera is even more resistant to HD5 then 19cPB alone. Additional studies using a higher concentration of HD5 (20 µM) confirmed the defensin-resistance of HAdV-19c, 19cF, and PB/GYAR (data not shown). Equivalent results were obtained using a FACS-based assay that requires 100-fold lower moi, indicating that variations in particle to pfu ratios among the virus preparations could not account for the differences in phenotype (data not shown). Studies equivalent to those in Figure 2B to measure HD5 binding to both 19cF and PB/GYAR did not detect a reduction in the amount of HD5 bound to these viruses compared to HAdV-5 (data not shown). Taken together, these studies demonstrate that HD5-mediated inhibition of HAdV infection is determined by species-specific sequences in the virus capsid and that critical neutralizing determinants are found in both fiber and penton base; however, the lack of reduction in overall HD5 binding to the resistant chimeric viruses suggests that additional, non-neutralizing binding determinants remain intact.

## Discussion

These studies extend our understanding of the mechanism of α-defensin-mediated neutralization of HAdV. We observed species-specific neutralization of HAdVs, which is dependent upon defensin binding to the virus capsid. Thousands of defensin molecules bind to each virus particle with an approximate K_D_ that correlates well with the IC_50_ for virus infection, and antiviral activity is dependent upon the tertiary structure of a correctly folded α-defensin molecule. Structural analysis by cryoEM indicates that defensins bind to all of the exposed major capsid proteins. Based on sequence analysis and cryoEM studies, we proposed that potential critical sites for defensin binding are located at the point of contact between penton base and fiber. The importance of these sites for defensin neutralization was confirmed by an analysis of virus chimeras comprised of sequences from sensitive and resistant HAdV serotypes, indicating that neutralization determinants are found in both fiber and penton base. In conjunction with our previous studies, this observation suggests a model in which defensin binding to these critical neutralization sites prevents fiber dissociation, thereby blocking subsequent steps in HAdV uncoating that are required for release of protein VI, endosomalysis, and infection.

Differential susceptibility to defensin was previously observed in studies of cutaneous and genital serotypes of HPV [9], suggesting that there are specific determinants on the HPV capsid that dictate defensin neutralization. To investigate whether this is also the case for HAdV, we tested the sensitivity of representative serotypes from 6 of the 7 HAdV species to defensins HD5 and HNP1. Consistent with more limited previous studies [12], we found that sensitivity to defensins is species specific. Because the defensin-capsid interaction is at least in part based on electrostatic interactions [13], a simple hypothesis is that defensin sensitivity would correlate with net hexon charge. However, this is not the case, even though the major electrostatic property of the AdV is from hexon [39]. This observation supports a model in which specific binding determinants dictate defensin sensitivity.

This conclusion is bolstered by the observation that only correctly folded HD5 has antiviral activity. Previous studies showed that the chemokine activity of some defensins is dependent upon defensin conformation [28]. α-defensin inhibition of bacterial toxins is also significantly reduced in defensin derivatives that cannot form disulfide bonds, as in the HD5-Abu used here [29], [40]. As HD5-Abu retains the same net positive charge as the correctly folded HD5, a purely charge-dependent mechanism cannot explain the neutralizing activity of this antimicrobial peptide. Similarly, although all natural defensins have a net positive charge, not all defensins (e.g., HBD-2) neutralize HAdV infection [8], [12], [13]. Therefore, these studies support the hypothesis that the HAdV capsid-defensin interaction is due to specific recognition of the virus capsid by defensins.

We used an equilibrium-binding assay to measure the affinity and stoichiometry of the defensin-capsid interaction. We found that as many as 2750 HD5 molecules are bound to each virus particle at saturation (V_max_) with an apparent affinity (K_D_) that approximates the IC_50_ for infection. The use of surface plasmon resonance to more accurately measure the capsid-defensin interaction would have been preferable for these studies; however, the large mass difference between HD5 (3.6 kDa) and the virus particle (150 MDa) precludes this approach. Although our analysis likely approximates the binding parameters of the system, there are some limitations. First, it is semi-quantitative because of the methods used to estimate both the number of virus particles in each sample and the amount of defensin bound. Second, there is no estimation of non-specific binding. Nonetheless, the data more closely fits a specific binding curve.

Our binding studies suggest one possible explanation for the enhancement of infectivity that is commonly observed for resistant serotypes. We observe specific binding of HD5 to the sensitive HAdV-5 and HAdV-7 serotypes but only a low level of binding to the resistant HAdV-19c, -25p, and -51p. Therefore, both neutralizing and non-neutralizing binding sites are likely present on the capsid. Defensin binding to non-neutralizing sites may neutralize electronegative surface charges and facilitate virus binding to the cell surface, functionally analogous to the enhancing effect of polybrene on retrovirus infection [41]. Consistent with this hypothesis, we showed that receptor-dependent and -independent binding of HAdV-5 to cells is enhanced by HD5, but not HD5-Abu, despite a complete block of productive infection. Moreover, mutation of critical neutralization determinants in the 19cF and PB/GYAR chimeras did not result in a noticeable reduction in overall HD5 binding. Thus, the capsid-defensin interaction is complex, and the presence or absence of critical neutralization determinants dictates the outcome (inhibition or enhancement).

The extensive difference density attributable to HD5 in our cryoEM analysis of Ad5.F35+HD5 is consistent with our estimated stoichiometry. HD5 binding sites were found on all of the major proteins of the capsid. Our studies do not address a physiologic role for hexon binding, although this binding may contribute to enhancement of infection due to charge neutralization. The accumulation of HD5 difference density was not equal among the four unique hexon positions in the asymmetric unit but rather was greatest on the peripentonal hexons. Since the possible binding sites presented by each hexon are equivalent, this may be due to multimerization of HD5 at the vertices, potentially creating bridges between the peripentonal hexons and the penton base. In crystal structures, α-defensins form dimers [42], [43]; however, it is unclear whether or not defensin dimerization plays a physiologic role. HD5 has been shown to form dimers and tetramers at concentrations below 5 µM, defensin self-association is greatly enhanced by binding to target proteins, and mutations that disrupt the ability of HD5 to form dimers also reduce target protein binding [40], [44]. Therefore, defensin dimerization or multimerization may also contribute to AdV binding and antiviral activity.

Similarly, we observed extensive binding of HD5 to the fiber. The fiber shaft was substantially thicker in the presence of HD5, and the fiber knob was poorly reconstructed. Either HD5 induces greater conformational flexibility in the fiber shaft leading to greater averaging of the fiber knob density, or HD5 affects the linker region between the shaft and the knob. Both the RGD loops and the fiber shaft contain multiple negatively charged residues that might serve as binding sites for the positively charged HD5 molecule. Nonetheless, the capacity of fiber to bind to CAR was not compromised by HD5 binding based on our previous studies showing HAdV-5 cell entry in the presence of HD5 and the observed reduction in cell binding of HAdV-5/HD5 in competition with 5FK [13], [36].

Our virus chimera studies support the existence of multiple binding determinants in the penton complex that are critical for neutralization. Disruption of a single binding determinant (e.g. DTET in fiber) is insufficient to completely abrogate neutralization. Rather, two or more sites must be simultaneously disrupted, as in the PB/GYAR chimera, to generate defensin resistance. Because resistance was also observed in the 19cF construct, at least two separate determinants are likely found in fiber. In each case, disruption of the neutralization sites led not only to resistance, but also to enhancement of infection. This finding supports the notion that enhancement and neutralization are competing processes mediated by defensin binding. Analysis of additional virus chimeras to map the neutralization determinants may provide a more detailed description of the binding sites important for inhibition and enhancement. They may also help explain the resistance of HAdV-41 to HD5 despite the presence of one acidic residue in the identified fiber neutralization determinant of both the short and long HAdV-41 fibers.

Based on our combined functional and structural studies, we propose a model for neutralization in which α-defensins bind to critical capsid determinants at the point of contact between fiber and penton base, thereby preventing fiber release. One implication of this model is that fiber dissociation is absolutely required for subsequent uncoating events. This model cannot distinguish between the dissociation of fiber and penton base from the capsid independently or together as a complex. In the first case, defensin may actively lock the fiber onto the penton base. Alternatively, HD5 may obstruct a conformational change in penton base that is required for its release with fiber still attached. Our studies provide strong support for a mechanism of neutralization of HAdV-5 by HD5 and, in combination with our previous report demonstrating stabilization of HAdV-5, -12, and -35 capsids by HD5 [13], suggest that other sensitive serotypes are neutralized by HD5 by a similar mechanism. However, detailed studies of additional HAdV/defensin combinations may reveal differences in the mechanisms.

Although the temporal order of uncoating events suggests that fiber release is a critical step [21], [22], no previous example of a specific inhibitor of this step leading to a block to infection has been described. Therefore, our studies not only provide insight into the mechanism of defensin-mediated neutralization of non-enveloped virus infection but also provide a new rationale for the design of entry inhibitors. In addition, our results shed further light on the earliest events of HAdV disassembly occurring during cell entry. Because other non-enveloped viruses (e.g., HPV) are also inhibited by defensins, studies of defensin neutralization may also provide insight into the entry mechanisms of these viruses.

The role of defensins *in vivo* against adenovirus or other non-enveloped viruses has not been demonstrated; however, several observations suggest that the neutralization model studied here could be relevant for antiviral immunity. HD5 concentration in the intestinal lumen has been estimated at 14–69 µM (50–250 µg/ml) [45], which is greater than that required to neutralize HAdV infection. Many HAdVs, including those that cause respiratory infections, have been shown to infect and replicate in the bowel and have been detected upon shedding in the feces. Thus, sensitive HAdV serotypes may encounter HD5 secreted by Paneth cells during natural infection. It is intriguing that HAdV-F serotypes, which cause primarily gastrointestinal infections, are resistant to HD5. The alpha-defensins of human neutrophils are found at high concentration in azurophil granules [46], [47]. Although measured at low concentrations in plasma, these molecules can be secreted or found in phagocytic vacuoles at high local concentrations (>10 mg/ml) [47]–[49]. These cells home to the site of infection where they could encounter HAdV in many tissues, including the ocular, oral, and pulmonary mucosa. AdVs have also been shown to interact directly with neutrophils and to be engulfed [50]. Additional studies are required to assess the role of defensins in antiviral immunity *in vivo*.

## Materials and Methods

### Cells, viruses, and peptides

Tissue culture reagents were obtained from Invitrogen (Carlsbad, CA). Human A549 cells (ATCC) were propagated in DMEM supplemented with 10% FBS. Stable 293 cells over-expressing the human β5 integrin subunit (293β5) were created by transfecting 293 cells (ATCC) with the human β5 gene (pCDNA3/β5, a gift from David Cheresh, University of California, San Diego; San Diego, CA). Transfected cells were selected for high integrin expression. Stable 293 cells over-expressing the V-protein of the paramyxovirus Simian virus 5 (293-SV5/V) were a gift of Kenneth Mellits (University of Nottingham, Loughborough, UK) [51].

HAdV-2p, -3p, -4p, -11p, -12p, -25p, -35p, -37p, -41p, and -51p were from ATCC. HAdV-7p and -14p were gifts of David Metzgar (Naval Health Research Center, San Diego, CA). HAdV-16p and -23p were gifts of Adriana Kajon (Lovelace Respiratory Research Institute, Albuquerque, NM). HAdV-19c was a gift of James Chodosh (Harvard Medical School, Boston, MA) [52]. The replication-defective HAdV-5 vector used in these studies (Ad5.eGFP) is E1/E3-deleted and contains a CMV promoter-driven enhanced green fluorescent protein (eGFP) reporter gene cassette. Ad5.F35 was constructed by replacing the entire fiber gene from a HAdV-5-based vector expressing β-galactosidase with that of HAdV-35p [53].

Virus chimeras were created by replacing the entire open reading frames of the HAdV-5 penton base or fiber with that of HAdV-19c by recombineering [54] in a BAC construct (pAd5-GFPn1) containing the entire genome of an E1/E3-deleted HAdV-5 vector expressing eGFP [55]. The GYAR and PB/GYAR constructs were created by replacing the codons for DTET in the HAdV-5 fiber gene with those for GYAR from HAdV-19c in the original pAd5-GFPn1 plasmid or in the previously constructed PB chimera plasmid, respectively. The fidelity of the chimera constructs was verified by sequencing the recombineered region and by restriction digest. To generate virus, 293β5 cells were transfected with the large Pac I restriction fragment of these BACs. Transfected cells were cultured until visible plaques formed. The identity of the final virus stock was confirmed by restriction digest. PCR was used to verify purity and absence of cross-contamination. The GYAR substitution in the fiber protein was confirmed by sequencing a PCR product from the final virus stock.

All wild type viruses were propagated in 293β5 or A549 cells except for HAdV-41p, which was propagated in 293-SV5/V. All AdV vectors were propagated in 293β5 cells. Cultures were infected with 300 particles/cell of purified viruses or from cleared lysates of original virus stocks. When complete cytopathic effect was observed, cells were harvested and concentrated by low speed centrifugation. For some serotypes, virus was precipitated from supernatant using 8% PEG [56]. Cell pellets were disrupted by three cycles of freezing and thawing. Mature virus was purified from the cleared lysate or PEG precipitate by two consecutive rounds of centrifugation [2–3 h at 111,000×g (avg.)] through continuous 15% to 40% CsCl gradients, dialyzed against three changes of A195 buffer [57], flash frozen in liquid nitrogen, and stored at −80°C.

Synthetic HNP1, HBD2, and HD5 were obtained from Peptides International, Inc. (Louisville, KY). HD5 derivatives containing the E14Q substitution or L-α-aminobutyric acid in place of cysteine were produced by solid phase chemical synthesis as described [25], [58]. The ribbon representation of HD5 (PDB 1ZMP) was generated with PyMOL [59]

### Infection assay

Prior to use in this assay, each virus stock was titrated on A549 cells. A virus concentration was chosen to produce 50–70% maximal signal in the absence of defensin as described below. To measure the effect of defensins on infectivity, purified virus was incubated with HD5 or HNP1 for 1 h on ice in serum-free DMEM (SFM). Confluent A549 cells in black wall, clear bottom 96-well plates were washed twice with SFM, and virus/defensin mixtures were added in a final volume of 35 µl/well. In parallel, wells were infected with two-fold serial dilutions of each virus to establish a standard curve for quantification with an upper limit of 200%. After 2 h, wells were washed twice and replaced with DMEM/10% FBS. Samples were incubated for approximately 48 h, fixed with paraformaldehyde, permeabilized in 20 mM glycine/0.5% Triton X-100 in PBS, and stained with an anti-hexon primary antibody (8C4, Fitzgerald Industries International, Acton, MA) and an Alexa Fluor 488-conjugated anti-mouse secondary antibody (Invitrogen, Carlsbad, CA). Plates were scanned for Alexa Fluor 488 signal using a Typhoon Trio variable mode imager (GE Healthcare, Piscataway, NJ). Total well fluorescence above background was quantified with ImageJ software [60]. For each virus, samples were quantified by nonlinear regression against the standard curve using Prism software (GraphPad Software, Inc., La Jolla, CA).

To test the activity of HD5 derivates, Ad5.eGFP was incubated with 15 µM of HD5, HD5-Abu, or HD5-E14Q. Infectivity was assessed by enumerating eGFP-positive cells by flow cytometry as described [13].

### Virus-defensin binding assay

To measure HD5 binding to HAdV-5 and -51, HD5 was serially diluted in PBS and mixed with 5 µg purified virus. After 1 h incubation on ice, one half of each sample was separated by ultracentrifugation [209,000×*g* (avg.) for 2 hrs at 4°C] on a discontinuous gradient consisting of 300 µl of 30% nycodenz and 200 µl of 80% nycodenz in 50 mM NaCl, 20 mM HEPES pH 7.4 using an SW55ti rotor with adaptors (Beckman Coulter, Inc.). The visible virus band was collected. The other half of each sample was used to make a standard curve for quantification. All samples were boiled in reducing loading buffer and separated using a 16% PAGEgel (Expedeon, Inc., San Diego, CA) or 10–20% Tris-Tricine gel (Bio-Rad, Hercules, CA). The gels were stained with Deep Purple (GE Healthcare) and imaged on a Typhoon Trio. Virus bands were quantified using ImageQuant NT software (GE Healthcare). The amount of HD5 in each sample was normalized to protein V and hexon. The amount of HD5 in the centrifuged samples was then quantified against the standard curve using Prism software. Affinity and stoichiometry were estimated from the average data of at least three independent experiments using Prism software.

### Fiber knob competition assay

Recombinant HAdV-5 fiber knob (5FK) comprising residues 387–581 of the HAdV-5 fiber and HAdV-16 fiber knob (16FK) comprising residues 151–353 of the HAdV-16 fiber, each containing an N-terminal hexahistidine tag, were expressed in BL21(DE3) cells (Invitrogen, Carlsbad, CA) and purified using TALON Metal Affinity Resin (Clontech, Palo Alto, CA) as previously described [61], [62]. Alexa Fluor 448 labeled-Ad5.eGFP [13] (4.2×10^9^ particles/sample) was incubated with or without 20 µM HD5 or HD5-Abu for 45 min on ice. In parallel, 1×10^5^ A549 cells in PBS+0.2% sodium azide were incubated with or without 200 nM 5FK or 16FK for 45 min on ice. The virus/defensin mixtures were combined with the cell/FK mixtures (final volume 100 µl/sample) and incubated for 45 min on ice. Samples were washed 2 times with cold PBS+1% FBS, fixed with 1% paraformaldehyde, and analyzed by flow cytometry for Alexa Fluor 488.

### CryoEM and image processing

Purified Ad5.F35 (160 µg/ml) was combined with HD5 (20 µM) and incubated for 45 min on ice. CryoEM grids were produced with an FEI Vitrobot. Electron micrographs were collected on an FEI Polara microscope (300 kV, FEG) operated at 300kV with the grids at liquid nitrogen temperature using the SAM semi-automatic data collection routine [63]. The defocus values of the micrographs ranged from 0.5 µm to 4 µm. The absolute magnification of the digital micrographs collected on a Gatan UltraScan 4000 (4000×4000 pixel) CCD camera was 397,878×, corresponding to a pixel size of 0.4 Å on the molecular scale. Individual particle images were selected from micrographs with in-house scripts and computationally binned to produce particle image stacks with various pixel sizes suitable for image processing (4.8 Å, 2.4 Å, and 1.6 Å). Particle images with a pixel size of 4.8 Å were used for initial CTF parameter determination with CTFFIND3 [64] and orientational parameter determination with FREALIGN [65]. A cryoEM structure of Ad5.F35 [31] was used as the starting three-dimensional model for FREALIGN refinement. Intermediate refinement rounds were performed using particle images with a 2.4 Å pixel and the final rounds of refinement were performed using particle images with a 1.6 Å pixel. Magnification refinement for the previously acquired Ad5.F35 and the new Ad5.F35+HD5 cryoEM particle images was performed together on a per particle basis in FREALIGN. Separate three-dimensional structures were generated for Ad5.F35 and for Ad5.F35+HD5 based on 3,040 and 2,611 particle images, respectively. The pixel size of the final structures was determined to be 1.61 Å by optimizing the agreement between the docked HAdV-5 hexon crystal structure (PDB 1P30) [32] and the cryoEM density maps with UCSF Chimera [66]. The resolution of the icosahedral capsid (radii 300–463 Å) of the Ad5.F35 reconstruction estimated by Fourier shell correlation is within the range of 6.9–5.3 Å; 6.9 Å (FSC 0.5 threshold); 6.1 Å (FSC 0.3); and 5.3 Å (FSC 0.143). The resolution of the icosahedral capsid of the Ad5.F35+HD5 reconstruction is 12.3–8.2 Å; 12.3 Å (FSC 0.5 threshold); 10.9 Å (FSC 0.3); and 8.2 Å (FSC 0.143). Both the Ad5.F35 and Ad5.F35+HD5 reconstructions were sharpened with a temperature factor of B = −450 Å^2^ and filtered to 12 Å resolution with cosine edge filtering using the BFACTOR program (http://emlab.rose2.brandeis.edu/software).

Difference mapping was performed by docking the HAdV-5 hexon (PDB 1P30) and HAdV-2 penton base/fiber N-terminal peptide crystal structure coordinates (1X9T) [33] within one facet of each reconstruction. The docked coordinates were converted to a density map with the pdb2mrc routine of EMAN v1.7 [67], filtered to 12 Å resolution, normalized, and subtracted from the Ad5.F35 and Ad5.F35+HD5 reconstructions.
